# Supplementary material for: Transcriptomic Insights into Mechanisms of Early Seed Maturation in the Garden Pea (Pisum sativum L.)
Source: Cells. 2020 Mar 23;9(3):779. doi: 10.3390/cells9030779 (PMC7140803; doi:10.3390/cells9030779)

Zhongwan−6,10(4)

Global Summary

%DE = 0.24

genes with fdr < 0.2 = 484 (223 + / 261 −)

genes with fdr < 0.1 = 427 (198 + / 229 −)

genes with fdr < 0.05 = 404 (187 + / 217 −)

genes with fdr < 0.01 = 358 (167 + / 191 −)

genes in genesets = 0

<FC> = 0

<t−score> = 0

<p−value> = 0

<fdr> = 0.76

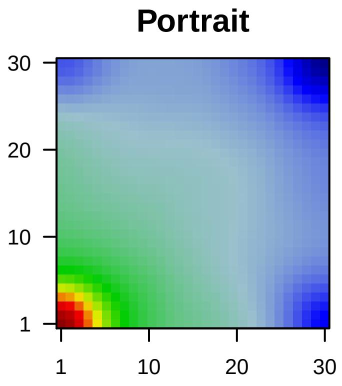

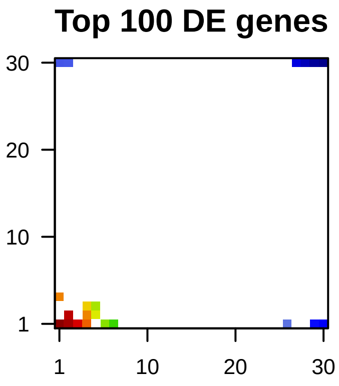

Global Genelist

Rank	ID	log(FC)	p−value	fdr	Metagene	Description
1	TRINITY_DN1	−1.01	2e−16	2e−15	30 x 1	
2	TRINITY_DN2	1.59	2e−16	2e−15	1 x 1	
3	TRINITY_DN1	1.35	2e−16	2e−15	1 x 1	
4	TRINITY_DN9	−0.73	2e−16	2e−15	30 x 30	
5	TRINITY_DN7	0.97	2e−16	2e−15	30 x 1	
6	TRINITY_DN7	−1.9	2e−16	2e−15	30 x 30	
7	TRINITY_DN5	−2.07	2e−16	2e−15	30 x 30	
8	TRINITY_DN5	1.36	2e−16	2e−15	1 x 1	
9	TRINITY_DN7	3.48	2e−16	2e−15	1 x 1	
10	TRINITY_DN4	5.77	2e−16	2e−15	1 x 1	
11	TRINITY_DN9	−0.82	2e−16	2e−15	30 x 1	
12	TRINITY_DN9	1.62	2e−16	2e−15	1 x 1	
13	TRINITY_DN2	−1.62	2e−16	2e−15	1 x 30	
14	TRINITY_DN2	−0.69	2e−16	2e−15	29 x 30	
15	TRINITY_DN2	2.07	2e−16	2e−15	1 x 1	
16	TRINITY_DN2	−1.17	2e−16	2e−15	30 x 1	
17	TRINITY_DN2	0.76	2e−16	2e−15	30 x 1	
18	TRINITY_DN2	−1.66	2e−16	2e−15	30 x 30	
19	TRINITY_DN2	1.19	2e−16	2e−15	1 x 1	
20	TRINITY_DN2	−0.67	2e−16	2e−15	30 x 30	

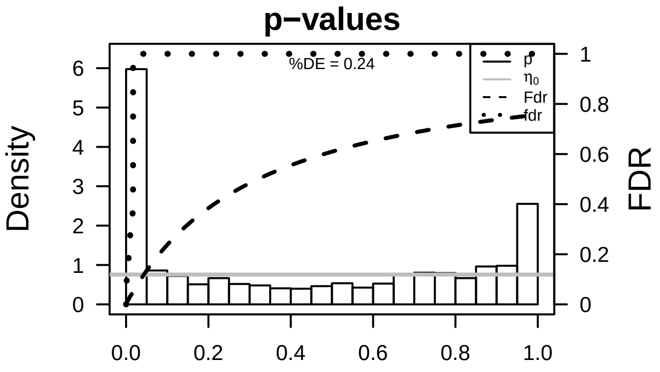

Supplement: Supplementary file 1 [file cells-09-00779-s001.zip › Supplementary materials/FileS4.zip!/FileS3/TFs - Results/Summary Sheets - Samples/Zhongwan.6.10.4..pdf]
